# Aquaporin Water Channels in the Mammary Gland: From Physiology to Pathophysiology and Neoplasia

**DOI:** 10.1007/s10911-013-9312-6

**Published:** 2013-12-13

**Authors:** Ali Mobasheri, Richard Barrett-Jolley

**Affiliations:** 1 School of Pharmacy, University of Bradford, Richmond Road, Bradford, BD7 1DP UK; 2 School of Life Sciences, University of Bradford, Richmond Road, Bradford, BD7 1DP UK; 3 School of Medicine, Faculty of Medicine and Health Sciences, Queen’s Medical Centre, Nottingham, NG7 2UH UK; 4 Center of Excellence in Genomic Medicine Research (CEGMR), King AbdulAziz University, Jeddah, 21589 Kingdom of Saudi Arabia; 5 Institute of Ageing and Chronic Disease, Faculty of Health & Life Sciences, University of Liverpool, Liverpool, L69 3GA UK

**Keywords:** Mammary gland, Milk production, Lactation, Aquaporin, Water channel, Immunohistochemistry, AQP1, AQP3, Neoplasia, Breast cancer

## Abstract

Aquaporins are membrane proteins that play fundamental roles in water and small solute transport across epithelial and endothelial barriers. Recent studies suggest that several aquaporin proteins are present in the mammary gland. Immunohistochemical techniques have confirmed the presence of aquaporin 1 (AQP1) and AQP3 water channels in rat, mouse, bovine and human mammary glands. Studies suggest that in addition to AQP1 and AQP3 AQP4, AQP5 and AQP7 proteins are expressed in different locations in the mammary gland. Aquaporins play key roles in tumor biology and are involved in cell growth, migration and formation of ascites via increased water permeability of micro-vessels. Emerging evidence suggests that expression of these proteins is altered in mammary tumors and in breast cancer cell lines although it is not yet clear whether this is a cause or a consequence of neoplastic development. This review analyzes the expression and potential functional roles of aquaporin water channels in the mammary gland. The physiological mechanisms involved in the transport of water and small solutes across mammary endothelial and epithelial barriers are discussed in the context of milk production and lactation. This paper also reviews papers from the recent cancer literature that implicate aquaporins in mammary neoplasia.

## Introduction

The mammary gland is a milk-producing organ that is characteristic of all female mammals and its overriding function is to synthesize and deliver milk to the newborn offspring. It is also present in a rudimentary and nonfunctional form in males. The mammary gland is a unique and dynamic organ that undergoes epithelial expansion and invasion during puberty and cycles of branching and lobular morphogenesis, secretory differentiation, and regression during pregnancy, lactation, and involution [1]. Embryonically, the mammary gland is derived from the ectoderm. Development begins with invasion of the underlying fat pad by a rudimentary ductal structure [2]. The epithelial nodules become buried in the mesenchyme, where they undergo differentiation under the influence of paracrine signals from the mesenchyme. These later develop into a tubuloalveolar structure that becomes functional in response to the hormonal changes associated with parturition and regulated by the endocrine system. Postnatal growth occurs in two phases: ductal growth and early alveolar development during estrous cycles, and cycles of cell proliferation, differentiation, and death that occur with each pregnancy, lactation, and involution.

**Fig. 1:**
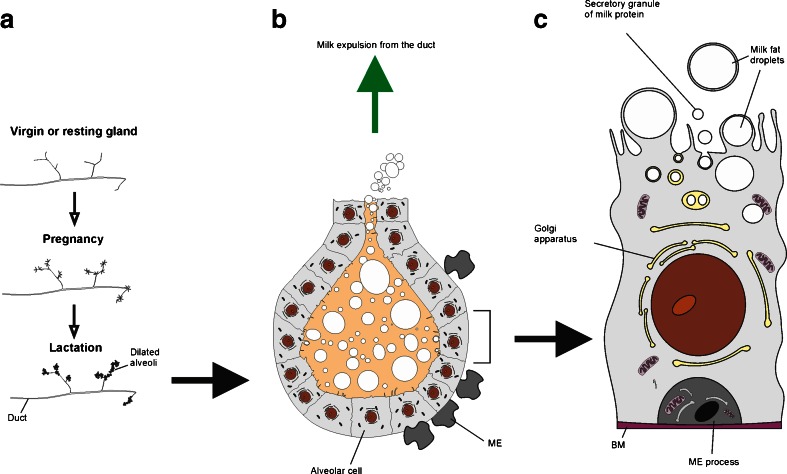
The mammary gland. **a** The growth of alveoli from the ducts of the mammary gland during pregnancy and lactation. Only a small part of the gland is shown. The “resting” gland contains a small amount of inactive glandular tissue embedded in a large amount of fatty connective tissue. During pregnancy an enormous proliferation of the glandular tissue takes place at the expense of the fatty connective tissue, with the secretory portions of the gland developing preferentially to create alveoli. **b** One of the milk-secreting alveoli with a basket of contractile myoepithelial cells (*green*) embracing it. **c** A single type of secretory alveolar cell produces both the milk proteins and the milk fat. The proteins are secreted in the normal way by exocytosis, while the fat is released as droplets surrounded by plasma membrane detached from the cell. Figure adapted and re-drawn from Molecular Biology of the Cell. (4th edition). Bruce Alberts, Dennis Bray, Julian Lewis, Martin Raff, Keith Roberts, and James D. Watson. New York: Garland Science; 2002

In terms of structure, the mammary gland is essentially similar to a modified sweat gland. In humans each fully developed breast is composed of 15–25 secretory lobes embedded in adipose tissue. Each secretory lobe is a compound tubular acinar gland. The acini lead to ducts, which are lined by cuboidal or columnar epithelial cells that are surrounded by myoepithelial cells. The ducts from each lobule empty into a lactiferous duct that leads to the nipple in the ampulla. The development, proliferation and differentiation of the mammary gland involve the concerted actions of a variety of hormones and growth factors including estrogen, progesterone, and prolactin. In addition to these regulatory endocrine factors, normal mammary development and lactation require cellular communication and cell-cell interactions between the stromal and parenchymal elements of the mammary gland [3]. Figure 1 outlines the growth of alveoli from the ducts of the mammary gland during pregnancy and lactation. This figure also highlights the differences between “resting”, “pregnancy” and “lactating” states and illustrates the functional unit of the mammary gland consisting of milk-secreting alveoli with a basket of contractile myoepithelial cells embracing it.

**Fig. 2:**
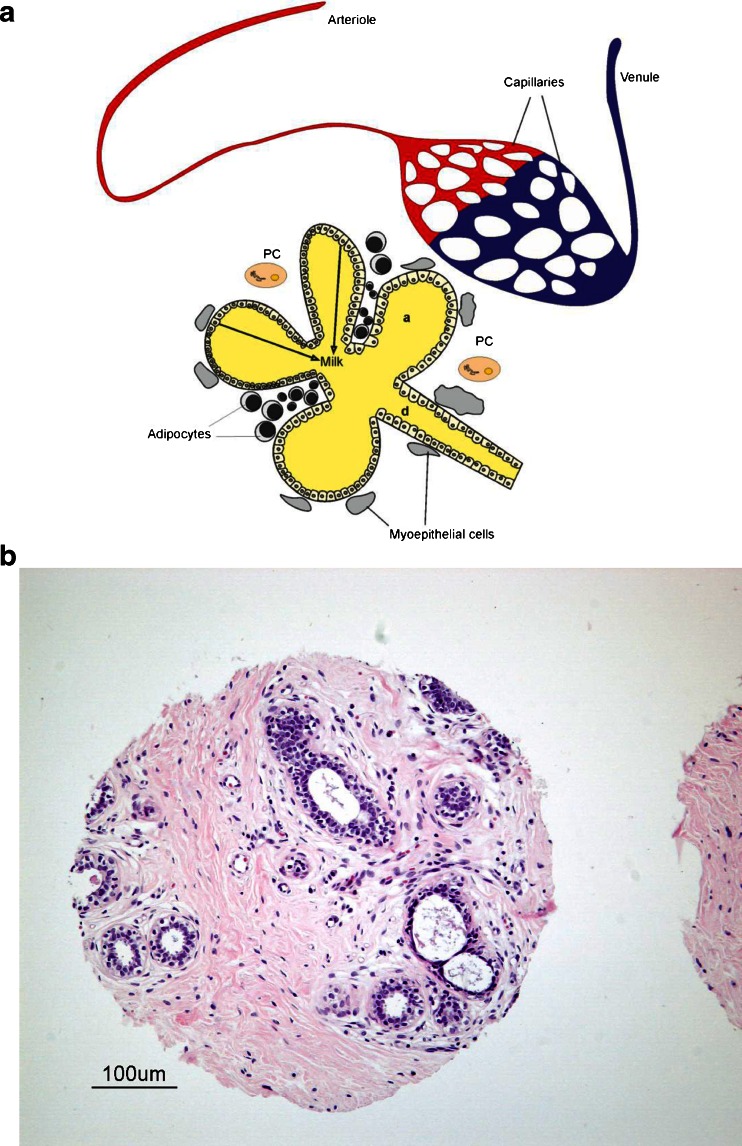
**a** Model alveolus (*a*) with subtending duct (*d*) showing blood supply, adipocyte stroma, myoepithelial cells, and plasma cells (*PC*). **b** Histology of human mammary gland. The sample shown in this figure is from a tissue microarray developed by the Cooperative Human Tissue Network (CHTN) of the National Cancer Institute (http://www.chtn.nci.nih.gov/). Mammary gland alveoli and ducts form an extensive network of interconnected structures

Mammary lobes are comprised of secretory acini, which are formed from cuboidal epithelial cells, responsible for the synthesis and secretion of milk, surrounded by myoepithelial cells. The latter contract during the milk ejection reflex, increase the pressure in acini and stimulate the expulsion of milk. Interstitial spaces between acini, ducts and lobes are filled with connective tissue and, for the most part, adipose tissue. In lactating animals, active acini secrete milk, which drains into small intralobular excretory ducts. Ducts are also lined with epithelial cells and may have an outer layer of myoepithelial cells derived from the same lineage [4, 5] (Fig. 2). Intralobular ducts join to form a network of interlobular ducts leading into a complex system of lactiferous ducts.

During pregnancy the alveolar duct epithelium proliferates, resulting in the formation of numerous secretory alveoli, which begin forming a protein-rich fluid called colostrum. The formation of milk is one of the key functions of the mammary gland. Milk consists of simple sugars, lipids, proteins, vitamins and minerals dissolved in water, which makes up to 88 % of its volume. The alveolar epithelial cells that synthesize milk are highly specialized, polarized and differentiated cells. Their function is to synthesize, package and export the key constituents of milk. At least five pathways are involved in the formation of milk in the tubuloalveolar structures of the mammary gland. The first, which is particularly relevant to the topic of this review article, is the secretion of monovalent cations and water. Current understanding is that water is secreted across the mammary epithelium in a transcellular manner, in response to an osmotic gradient produced largely by the lactose content of the milk [6, 7]. Water is essentially drawn across the alveolar cells by the concentration gradient created by osmotically active milk sugars. This process is followed by the transport of immunoglobulins, milk lipids, milk proteins and other buffers and electrolytes. The processes involved in milk production are discussed in the following section.

## Milk Production

**Fig. 3:**
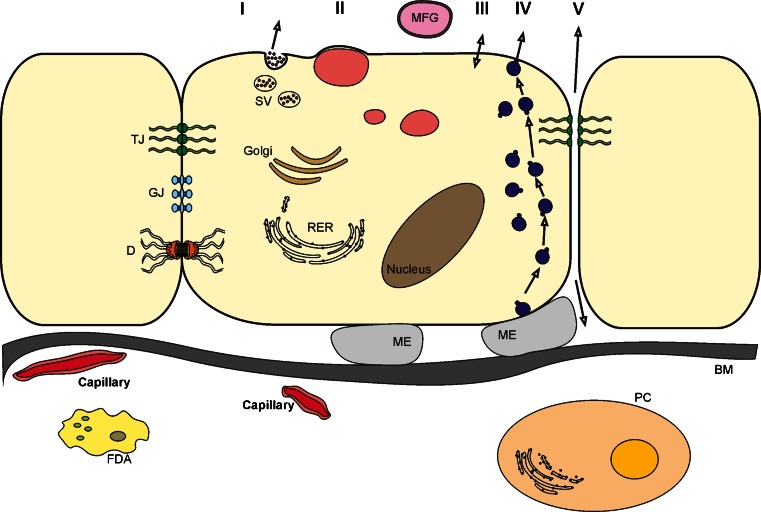
Alveolar cell from lactating mammary gland. *TJ* tight junction; *GJ* gap junction; *D* desmosome; *SV* secretory vesicle; *FDA* fat-depleted adipocyte; *PC* Plasma Cell; *BM* basement membrane; *ME* cross section through process of myoepithelial cell; *RER* rough endoplasmic reticulum. See text for explanation of secretory pathways *I* (exocytosis), *II* (lipid), *III* (apical transport), *IV* (transcytosis) and *V* (paracellular pathway)

Five distinct processes are involved in the mammary epithelium in the secretion of milk (Fig. 3). These pathways operate in parallel to transform precursors derived from the blood or interstitial fluid into milk constituents. Although the biochemical processes involved are fundamentally the same in all mammals, differences in their relative rates and, in some cases, in the nature of the products synthesized result in milks whose composition differs widely from one species to another. Some of the milk secretion pathways, e.g., exocytosis of protein-containing vesicles and transcytosis of immunoglobulins, are similar to processes in many exocrine organs. In contrast, the mechanism for fat secretion is unique to the mammary gland. Four secretory processes are synchronized in the mammary epithelial cell of the lactating mammary gland: exocytosis, lipid synthesis and secretion, transmembrane secretion of ions and water and transcytosis of extra-alveolar proteins such as immunoglobulins, hormones and albumin from the interstitial space. A fifth pathway, the paracellular pathway, allows the direct transfer of materials between the milk space and the interstitial space. This pathway is open in the pregnant gland and allows the transfer of molecules at least as large as intact immunoglobulins. It is closed in the fully lactating gland providing a tight barrier between the milk and interstitial spaces. This barrier opens again in the presence of mastitis and during involution [8, 9].

## Breast Cancer

Breast cancer refers to tumors that form in tissues of the mammary gland. The most common type of breast cancer is ductal carcinoma, which begins in the lining of the milk ducts. Another common type of breast cancer is lobular carcinoma, which occurs in the lobules of the breast. Invasive breast cancer refers to metastatic cancer that has spread from breast ducts or lobules to surrounding normal tissues. The global burden of breast cancer exceeds all other cancers and the incidence rates of breast cancer are constantly increasing. Breast cancer is the second most common cancer worldwide after lung cancer, the fifth most common cause of cancer death, and the leading cause of cancer death in women. Although breast cancer affects both men and women, the incidence of male breast cancer is very rare. Up-to-date statistics from the National Institutes of Health suggest that in the United States there were 232,340 (female) and 2,240 (male) new cases resulting in 39,620 deaths in females and 410 in males.

This article does not focus on the usual suspects in breast cancer. We do not discuss the recent data on BRCA mutations, the expression of HER2, the estrogen receptor, the progesterone receptor or new adjuvant therapies. The aim of this article is to discuss the role of aquaporins in mammary gland physiology and review the recent information on aquaporins in cancer.

## Aquaporin Water Channels

The fact that water has the ability to cross the hydrophobic membrane and enter (and leave cells) had been known for decades, but the mechanism was somewhat of a paradox. The paradox was solved by the discovery of a family of 28 kDa membrane transporters with high permeability for water [10]. The first of these proteins to be discovered was initially called “CHIP28” but is now known as aquaporin 1 (AQP1) [10, 11]. Transfection of cells with AQP1 increased membrane permeability to water by 50x. The discovery of aquaporins led to the award of the Nobel Prize in chemistry to Dr. Peter Agre in 2003.^1^^,^ 2 A transcript of his Nobel lecture was published in Bioscience Reports in 2004 [12]. Following the initial discovery a number of advancements have been made in the field. Aquaporins are a family of membrane bound proteins that are believed to be ubiquitously expressed in cellular and intracellular membranes. They are extensively distributed in microorganisms [13], animals [14–16] and plants [17–19]. They are small transmembrane proteins that are expressed in a variety of epithelial tissues where they are responsible for regulating rapid water movement across epithelial barriers driven by osmotic gradients. Initially, the main role of aquaporins was believed to be water and small solute transport across epithelial and endothelial barriers [20, 21]. It is now known that aquaporins are found in a wide range of non-epithelial/endothelial cells from acinar cells to chondrocytes [22–24], leukocytes to astrocytes, and in a wide range of reproductive organs [25–27]. Thus aquaporins appear to be present in most, if not all, cell types. Furthermore, aquaporins serve important functions in a wide range of processes such as cell division, cell migration, cellular volume regulation and apoptosis. These are clearly of importance when considering the pathogenesis of carcinomas. Despite this ubiquitous expression and fundamental importance in cell biology, genetic deletion of AQP genes has a less dramatic effect on animal survival than may be expected. Transgenic mice lacking AQP1 water channels survive but they have severely impaired urinary concentrating ability [28] and lack of a functional AQP2 gene leads to a rare form of nephrogenic diabetes insipidus [29]. Survival in the absence of specific aquaporins may be because of the phenotypic adaption that takes place; for example, genetic knock-out of AQP1 leads to up-regulation of other aquaporins such as AQP4, AQP7 and AQP8, at least in some tissues [30]. Mammalian aquaporins are located at strategic membrane sites in endothelia and a variety of epithelia, most of which have well-defined physiological functions in fluid absorption or secretion [31]. To date, 13 members of the aquaporin gene family have been identified in humans: AQP0-AQP12 [32]. Animal genome projects have also confirmed the presence of multiple aquaporin genes encoding distinct protein isoforms. The proteins encoded by aquaporin genes have been classified into two major groups based on their substrate permeabilities: 1) the classical water permeable aquaporins are permeated by water and include AQP1, AQP2, AQP4, AQP5 and AQP8 [14]; 2) the water and small solute permeable aquaglyceroporins exhibit permeability to water and a range of small neutral solutes including substances such as glycerol and urea. Aquaglyceroporins include AQP3, AQP7, AQP9 and AQP10 [14, 33]. Several aquaporins, including (AQP1, AQP4, AQP5 and AQP8) have also been proposed to facilitate entry of gases such as CO_2_, NO and ammonia to cells, but this important topic is discussed and reviewed elsewhere [34, 35]. The more controversial suggestion that some aquaporins (AQP1 for example) conduct small cations (e.g., Na^+^ and K^+^ [36, 37]) is disputed by the groups’ of Agre and Pohl [34, 38].

## Hormone Regulation of Aquaporins

The physiological advantage of membrane expression of ion channel and porin proteins compared to the evolution of “leaky” membranes is that membrane permeability can be precisely and dynamically controlled by variations in protein expression and activity. This allows for changing membrane water permeability in response to changing osmotic environment or the physiological need. This is achieved in two ways, firstly the permeability of the aquaporin channels themselves is variable, being strongly dependent on the molecular weight of the osmolytes in the aqueous solution to which the channel is exposed [39]. Secondly, functional expression can be dynamically regulated. Although little data exists on this in mammary gland tissue, there are several examples of this phenomenon elsewhere. The most commonly observed example is in the kidney collecting duct water (principle cell) where permeability increase is induced by arginine-vasopressin induced phosophorylation via V2-R receptors [40–43]. This system is a key step in regulation of re-absorption and thus urinary volume control and the regulation of body water balance. The cellular mechanism is understood in some detail and involves the translocation of protein to the cellular membrane, a mechanism, which is significantly faster than protein synthetic generation of new protein de novo. During brain edema and in joint diseases such as osteoarthritis, synovitis and rheumatoid arthritis aquaporin expression changes as an apparent adaptation to changes in the local osmotic environment [24, 44–46]. Furthermore, steroid hormones increase AQP1 expression in both peribronchiolar and uterine vascular endothelial tissues as a part of tissue fluid management [47, 48]. We will also discuss, below, how AQP1 is thought to be involved in estrogen mediated angiogenesis in the mammary gland and outline how increases in AQP3 expression promote FGF-2 stimulated migration of breast cancer cell lines.

## Immunolocalization of Aquaporins in Rat, Mouse, Human and Bovine Mammary Glands

Until recently nothing was known about the expression of aquaporins in the mammary glands of rodents, humans and dairy animals. Recent immunohistochemical studies of aquaporins in the rat and mouse mammary gland have confirmed the presence of AQP1 and AQP3 proteins in both species [49]. Matsuzaki and co-workers also used RT-PCR to study the expression of AQP1, AQP2, AQP3, AQP4, AQP5, AQP6 and AQP7 in the rat mammary gland [49]. In addition to AQP1 and AQP3 they found evidence for the presence of AQP4, AQP5, AQP7 and AQP9 transcripts in the rat mammary gland. However, they did not use antibodies to localize these proteins to specific tissues within the breast.

**Fig. 4:**
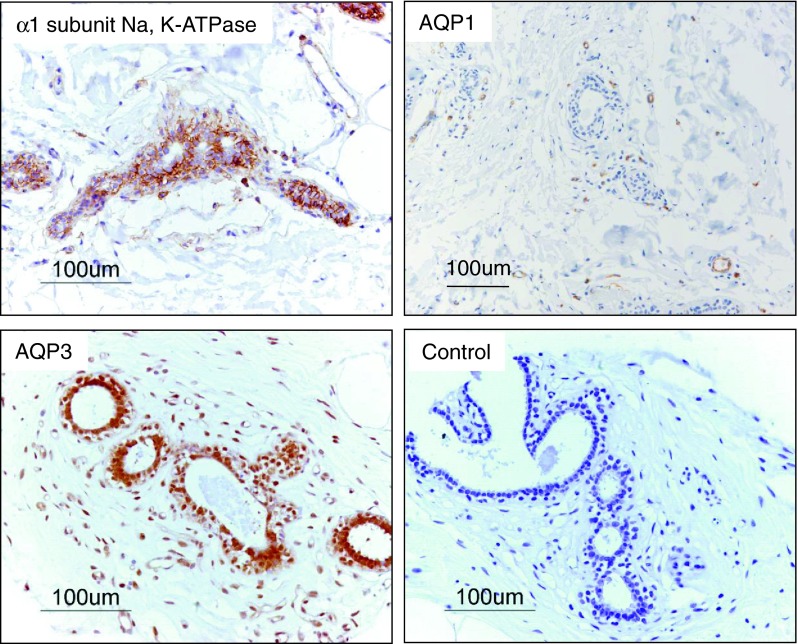
Expression of the α1 subunit of Na, K-ATPase, AQP1, AQP3 in normal human breast samples represented on a CHTN human tissue microarray

Work using human tissue microarrays has provided direct immunohistochemical evidence for the presence of AQP1 and AQP3 in human mammary glands [41, 50] (Fig. 4). AQP1 has been located in both the apical and basolateral membranes of capillary endothelia in the rodent mammary gland [49]. AQP3 has been located immunohistochemically in basolateral membranes of secretory epithelial cells and intralobular and interlobular duct epithelial cells in rat and mouse mammary tissue [49]. Although the expression of mRNA transcripts encoding AQP4, AQP5, AQP7 and AQP9 was demonstrated in rodent mammary by RT-PCR, the presence of the corresponding proteins was not investigated and the presence of aquaporins 4, 5 and 7 in the rodent mammary gland was not studied using immunohistochemical methods [49].

## Physiological Relevance of Aquaporins to Milk Production

Milk is a secreted body fluid that consists of water plus lipids, electrolytes, vitamins, sugars and specific milk proteins. The delivery of water to the mammary gland by the circulatory system and the movement of water across endothelial and epithelial barriers are critical for milk synthesis and secretion in lactating animals. There is published information on sodium and chloride transport in mammary epithelia [51]. However, the functional anatomy of the mammary gland has not been extensively studied in the context of water transport across endothelial and epithelial barriers and bulk fluid movement in lactating animals. Consequently, very little published information is available; AQP1 and AQP3 proteins have been shown to be present in the capillary endothelia and mammary epithelial cells, respectively [49]. Similar observations suggest that AQP1 and AQP3 proteins are also present in non-lactating human mammary glands [41, 50]. Data recently published on aquaporins in the bovine mammary gland [52] confirm some of the recent observations made by [49] in rodent mammary glands and similar observations in human mammary glands [41, 50]. Bovine data [52] also confirm the presence of these proteins in the bovine mammary gland, although in distinct cellular locations. A striking and previously unreported expression of AQP1, at high abundance, is also observable in myoepithelial cells underlying teat duct epithelia [52]. The physiological reason for this is at present unknown, but high AQP1 levels in this location may contribute to increased permeability of teat duct epithelia in the lactating bovine mammary gland. The relative abundance of the protein also appears to represent a distinct aspect of differentiation of myoepithelial cells in this location, compared with those surrounding the acini.

Goats’ milk is known to contain numerous cell fragments known as “christiesomes” which originate from secretory epithelial cells of the mammary gland [53, 54]. These cell fragments are known to contain intact and well-preserved endoplasmic reticulum, mitochondria and lipid droplets. Furthermore, they are capable of triglyceride synthesis. Although cows’ milk has been shown to contain very few cellular fragments, it does contain different and denser particles containing fewer vesicles and numerous microvillus-like protrusions on one side—known as “sunbursts” [53]. Although it has been suggested that these particles are residues of dead cells, it is possible that these fragmented cytoplasm-containing entities contain membrane proteins, which are targeted to the apical membranes of mammary secretory epithelial cells. Recent studies have provided evidence for urinary excretion of AQP2 water channel proteins during pregnancy [55, 56]. Biochemical analysis of “christiesomes” and “sunbursts” should reveal if these cellular fragments contain membrane proteins of the apical membranes of alveolar epithelial cells. Comparing this data with published data from rodent studies clearly indicates that some of the aquaporins expressed in the lactating mammary tissues were found in expected anatomical locations; immunohistochemical labeling in the rat mammary gland suggests that AQP1 is localized to the capillaries and AQP3 is localized to the basolateral membranes of the alveolar secretory cells. These results suggest that aquaporins are present in lactating mammary glands and may be participants in the control of milk water content by diluting the sugar, protein and lipid contents of milk to an isotonic solution as it descends through the teat duct system. This data has provided interesting new information about the possible regulation of water homeostasis in the mammary gland. Milk yield is of major economic importance to the dairy industry. Therefore, it is necessary to understand the underlying molecular physiology involved in fluid movement in lactation.

## Aquaporins and Cancer

Aquaporins are more than just passive water and small solute channels in biological membranes [57]. They are actively regulated and increasingly implicated in a number of important clinical disease states [58]. Recent evidence suggests that aquaporins are also involved in cell migration [59], angiogenesis [60], and tumor growth [61]. These water channel proteins are strongly expressed in tumor cells of different origins, particularly aggressive tumors [62]. AQP1 is ubiquitously expressed in endothelia of many human tissues [41]. Immunohistochemical and histomorphometric studies have confirmed the presence of AQP1 in endothelial barriers of almost all human tissues as well as many epithelial barriers involved in absorptive and secretory functions [41]. AQP1 is also present in tumor vascular endothelium [62]. Interestingly, targeted AQP1 gene disruption impairs angiogenesis and cell migration and AQP1-null mice show defective tumor angiogenesis resulting from impaired endothelial cell migration [62, 63]. AQP-expressing cancer cells show enhanced migration in vitro and greater local tumor invasion, tumor cell extravasation, and metastases in vivo than AQP1-null transgenic mice [62]. AQP-dependent cell migration may involve AQP-facilitated water influx into lamellipodia at the front edge of migrating cells [63]. High throughput studies using tissue microarrays (TMAs) have shown that AQP1 is an excellent marker of microvasculature but it is heterogeneously expressed in different human tumors and is not necessarily expressed in all neoplastic cells [64]. It has been proposed that increased AQP1 expression in some human adenocarcinomas may be a consequence of angiogenesis and important for the formation or clearance of tumor edema [64]. There is growing data in the literature concerning the involvement of AQP1 and AQP4 in human brain tumor growth and edema formation [61, 65]. These studies suggest that the presence of AQP1, but not AQP4, enhances glioma growth and migration, while AQP4 enhances cell adhesion highlighting differential biological roles for AQP1 and AQP4 in gliomas [66].

Aquaporins have therefore been proposed as novel pharmacological targets in cancer and associated edematous states [67]. Potentially, block of aquaporins could limit angiogenesis and inhibit tumor growth and metastasis. Therefore it has been suggested that novel therapeutic strategies approaches may be developed by antagonizing their biological activity [65]. It should be noted that pharmacological inhibition of aquaporins could have a greater protective effect than that resulting from genetic deletion, because there is less opportunity for adult cells to exhibit the phenotypic switch of aquaporin expression discussed above in APQ1 knock-out studies. Consequently, there is growing interest in aquaporin-based diagnostics [68] and the development of small-molecule aquaporin modulators for treating various clinical states including brain edema, neuro-inflammation, glaucoma, epilepsy, cancer, pain, and obesity [58].

## Aquaporins in Breast Cancer

Studies on the role of CCAAT/enhancer binding protein (C/EBP) family of bZIP transcription factors (particularly C/EBPβ) in mammary gland development and breast cancer have shown that targeted deletion of C/EBPβ isoforms results in severe inhibition of lobuloalveolar development, blocks functional differentiation, and induces changes in ductal morphogenesis. The loss of C/EBPβ isoforms results in altered expression of a number of membrane proteins, transporters and molecular markers, including the progesterone, estrogen, prolactin receptors and several transporter proteins (NKCC1 and AQP5) [69]. This was one of the early studies that implicated AQP5 in mammary gland development [69]. Further studies investigated the distribution of AQP1 in tumors of the prostate, colon, lung, breast and ovary using TARP multi-tumor Tissue TMAs and CHTN (Cooperative Human Tissue Network) TMAs [64]. AQP1 expression was higher in advanced mammary and colorectal carcinomas where AQP1 immunoreactivity was also seen in some neoplastic tumor cells [64]. Subsequent studies from our laboratory investigated expression of AQP1, the GLUT1 glucose transporter and Na, K-ATPase in canine mammary glands and mammary tumors [70]. We investigated the expression of these proteins in normal canine mammary glands and in benign and malignant mammary tumors, using immunohistochemistry and semi-quantitative histomorphometry. Interestingly, AQP1 immunoreactivity was absent from the majority of canine tumor specimens studied. However, this could have been due to the lack of antibody cross-reactivity. The antibodies used in the study were raised against rat AQP1 and could have cross-reacted poorly with their with canine AQP1 counterparts [70].

Otterbach and co-workers used immunohistochemical techniques to investigate the expression of AQP1 in 203 invasive breast carcinomas with long-term follow up data [71]. AQP1 expression was seen in 11 tumors (5.4 %) and showed a highly significant correlation with high tumor grade, medullary-like histology, “triple-negativity”, cytokeratin 14 and smooth muscle actin expression. The authors used univariate analysis to show that AQP1 was significantly associated with poor prognosis. They also used multivariate analysis to demonstrate that AQP1 protein expression is an independent prognostic marker if the tumors are properly stratified by age, tumor size, lymph node status, histological grade, ER status and CMF therapy. Based on these results the investigators suggested that AQP1 expression is a characteristic feature of aggressive basal-like breast carcinomas [71].

**Fig. 5:**
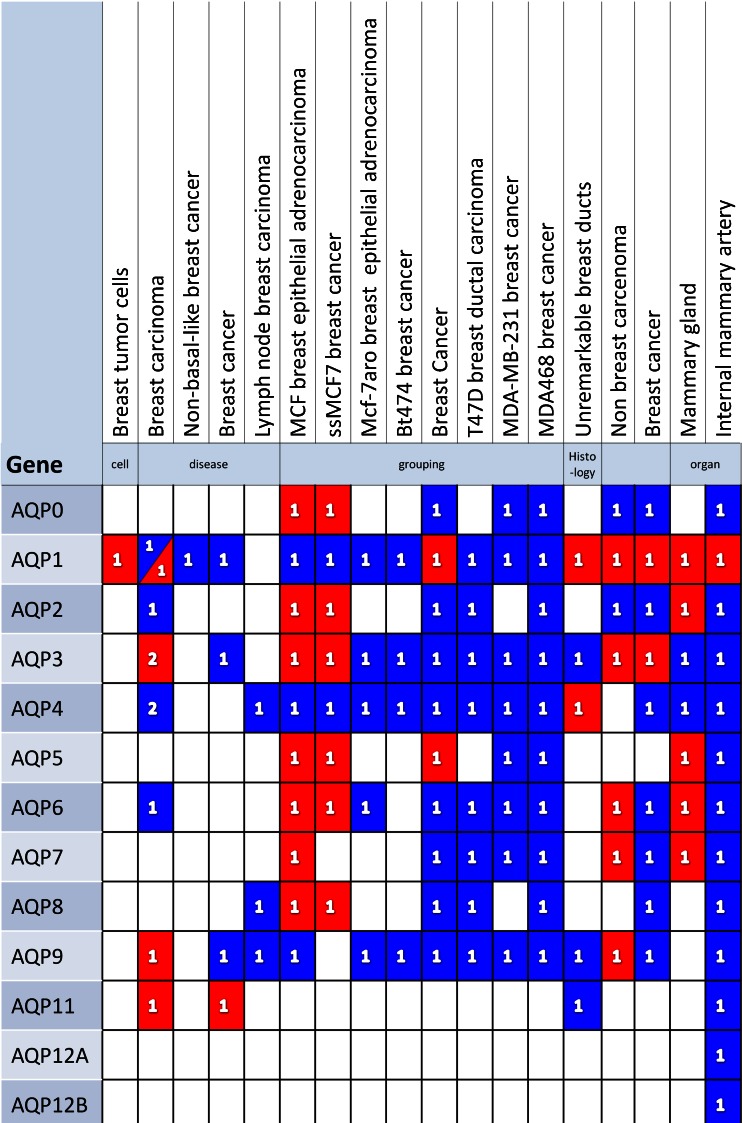
Differential expression of AQPs in human mammary glands, breast tissues and cell lines. We analysed the aquaporin gene expression patterns using the Gene Expression Atlas (GXA: http://www.ebi.ac.uk/gxa) as described previously [72]. The latest release 2.0.21.3 (Ensembl version 72) comprising a total of 2,841 experiments, 88,280 assays and 16,398 conditions. The database hosts publicly available gene expression data from the ArrayExpress archive. The database was queried for all aquaporin ‘genes’ that are differentially expressed breast tissue. The analysis was restricted to Homo sapiens. Each row is an AQP gene and each column is a condition. The expression patterns are denoted by *blue* or *red color*. Blue indicates under expression and red indicated over expression of a particular AQP gene. Note that non-differentially expressed results are not included.Number inside the square or triangle indicates that the number of experiments where the particular AQP gene was under or over expressed.

No genome wide transcriptomic studies have specifically investigated changes in aquaporins in breast cancer, however, most of the common microarray RNA chips, including Affymetrix, Agilent and Illumina include multiple probe sets for the aquaporins. Analysis over a range of different breast cancer cell lines and experiments listed in the Gene Expression Atlas [72] reveals changes in transcript abundance of many of the aquaporins (see Fig. 5). Many of these are decreased in expression in cancerous tissue, perhaps revealing that some of the observed increases in aquaporins protein expression could result from post-translational changes. Casey et al. [73] performed a large microarray analysis of genome-wide expression in normal (breast reduction) and breast cancer patients. Both epithelial tumor and stromal tumor samples were analyzed using the popular Affymetrix Human Genome U133A 2.0 Array. The raw and processed data are publically available in the Gene Expression Atlas (E-GEOD10797) and the Gene Expression Omnibus database (Accession no. GSE10797: http://www.ncbi.nlm.nih.gov/geo/geo2r/). Expression data is presented for many of the possible aquaporins isoforms (AQP1, AQP2, AQP3, AQP4, AQP5, AQP6, AQP7, AQP8 and AQP9). There is considerable variation between individual patients, with some showing very large increases in some isoforms of channel. The most notable observation, however, is the differences between epithelial and stromal tumor tissue; whilst there are no significant differences between any of the aquaporin transcripts in normal breast tissue; AQP1, AQP8, AQP4, AQP6 and AQP9 are all significantly higher in stromal tumor tissue, implying a considerable change in cellular phenotype. There is no evidence from this transcriptomic data of a phenotypic switch from one isoform to another to that observed with genetic deletion [30].

As discussed earlier, AQP1 is a well-established marker of microvessels. AQP1 has been proposed as a mediator of estrogen-induced angiogenesis in human breast cancer. A recent study has shown that AQP1 plays a crucial role in estrogen-induced tubulogenesis of vascular endothelial cells [60] providing fresh new insight into the molecular mechanisms underpinning the angiogenic effects of estrogen. The authors found that expression of AQP1 in blood vessels of human breast carcinoma tissues were significantly higher than controls [60], confirming the observations of several earlier studies [64, 71].

A genetic study in Germany investigated the prognostic relevance of the AQP5 -1364C>A polymorphism in primary breast cancer [74]. Samples from a total of 107 patients with early stage breast cancer were analyzed in order to test the hypothesis that this polymorphism is associated with lymphogenous and hematogenous tumor cell dissemination and overall survival. SNPs in AQP5 were not found to be associated with hematogenous or lymphogenous tumor cell spread. However, there was an association between SNPs in AQP5 and progesterone receptor positivity, which might have implications for future adjuvant treatment [74].

A study conducted in Nanjing, China screened the expression profile of AQP0-12 in breast cancer tissues and corresponding normal tissues by using RT-PCR, western blotting and immunohistochemistry [75]. AQP1, AQP3, AQP4 and AQP5 exhibited differential expression compared to normal tissue. Interestingly, this study found that AQP5 was expressed mainly in cell membranes of mammary carcinomas, with AQP5 being not detectable in normal breast tissues. Furthermore, expression of AQP5 was found to be associated with cellular differentiation, lymph node invasion, and tumor staging [75].

Subsequent studies on breast cancer cell lines have shown that AQP5 is a marker protein for proliferation and migration of human breast cancer cells [76]. Jung and co-workers detected AQP5 mRNA and protein in the human breast cancer cell lines MCF7 and MDA-MB-231 by RT-PCR and immunoblotting. Immunolabeling of AQP5 was seen in ductal epithelial cells of human breast tissues but the apical polarity of AQP5 in ducts was lost. The prominent expression of AQP5 and the loss of polarity of ductal epithelial cells were associated with the progression of breast carcinoma. The authors concluded that AQP5 over-expression plays a role in cell growth and metastasis in human breast cancer [76].

Several studies have also reported on the expression and functional role of AQP3 in breast cancer cell lines. A very recent study has shown that AQP3 is required for FGF-2-induced migration of human breast cancers [77]. The authors used MDA-MB-231 and Bcap-37 cell lines as models and inhibited the expression of AQP3 expression using lentiviral constructs that stably express shRNA against the mRNA encoding AQP3. Stimulation with FGF-2 treatment increased AQP3 expression and induced cell migration. Silencing AQP3 expression inhibited FGF-2 induced cell migration. The study concluded that AQP3 is required for FGF-2-induced cell migration in cultured human breast cancer cells. These findings also highlight the importance of FGFR-PI3K and FGFR-ERK signaling in FGF-2-induced AQP3 expression.

Finally, Trigueros-Motos et al., [78] used the capecitabine catabolite 5′-deoxy-5-fluorouridine (5′-DFUR) (a nucleoside analog used in the chemotherapy of solid tumors) to demonstrate that AQP3 is required for cytotoxic activity of 5′-DFUR in the breast cancer cell line MCF7 and that this aquaporin is implicated in cell volume increase and cell cycle arrest. Thus, it would appear that AQP3 is more than a marker and a passive channel. Its function is actually important for chemotherapy.

## Conclusions

The mammary gland remains an enigma. The distinctive developmental aspects and complex regulation by hormones and growth factors has made the study of the mammary gland function a major focus of research for scientists from many different biological and biomedical disciplines. The increased incidence of breast cancer is another important reason for studying the mammary gland. A number of studies have attempted to identify specific genes required for the functional development of mammary epithelium [79]. However, very few studies have examined the potential contribution of aquaporins to milk production. The altered expression of a number of molecular markers, including the progesterone, estrogen, and prolactin receptors, the Na/K/2Cl cotransporter proteins (NKCC1) and aquaporin 5 (AQP5), and several markers of skin differentiation (Sprr2A and keratin 6) has been reported in breast cancer [69]. The identification of AQP5 expression in neoplastic breast epithelium preceded subsequent reports of other aquaporins in rat, mouse [49] and bovine mammary glands [52]. The studies, cited in this review article, suggest that several aquaporin proteins collaborate in the production of milk in the mammary gland. There is also emerging evidence to suggest that aquaporins play important roles in breast cancer development.

From a physiological perspective, future studies will need to determine if additional aquaporins are involved at different stages of lactation and correlate the expression of these with the expression of milk protein genes and proteins. A better understanding of the molecular mechanism involved in milk production will have significant benefits for animal breeding programmes.

From a pathophysiological perspective, there is an almost universal observation that AQP protein expression increases in cancer. The biological implications of this are not really completely clear but the information available supports the hypothesis that aquaporins are implicated in neoplastic transformation in the breast and other organs. This may be due to the involvement of aquaporins in angiogenesis or cell migration, but an additional intriguing possibility is that aquagylceroporins, in particular, could be coupled to the cellular metabolism of tumor-committed cells. Entry of glycerol through channels such as AQP3 can increase availability of intracellular ATP in some cell types [80] and therefore its inhibition could therefore slow proliferation. The role of aquaglyceroporins in the mammary gland should be considered in the context of lactation and neoplastic transformation. The role of aquaglyceroporins may not be limited to cell proliferation. The entry of glycerol into mammary epithelial cells is also likely to be important for lipogenesis which is equally important for mammary gland health and disease.

Further work is required to determine whether aquaporins are viable therapeutic targets or reliable diagnostic and prognostic biomarkers, understand the functional roles of aquaporins in breast cancer and determine whether these proteins can be targeted in new anti-cancer therapies.

## Abbreviations

5’-DFUR: 5’-deoxy-5-fluorouridine
AQP: Aquaporin
ATP: Adenosine triphosphate
BRCA: Breast cancer susceptibility protein
bZIP: Basic leucine zipper domain
C/EBP: CCAAT/enhancer binding protein
CHIP28: Channel-forming integral protein 28
CHTN: Cooperative Human Tissue Network
CMF: Cyclophosphamide methotrexate fluorouracil
CO_2_: Carbon dioxide
ER: Estrogen receptor
FGF-2: Fibroblast growth factor 2
FGFR-ERK: Fibroblast growth factor receptor-extracellular signal-regulated kinase
FGFR-PI3K: Fibroblast growth factor receptor-phosphatidylinositide 3-kinase
GLUT1: Glucose transporter 1
HER2: Human epidermal growth factor receptor 2
MCF-7: Michigan Cancer Foundation-7
Na, K-ATPase: Sodium-potassium-ATPase
NKCC1: Na/K/2Cl cotransporter
NO: Nitric oxide
RT-PCR: Reverse transcription polymerase chain reaction
SNP: Single-nucleotide polymorphism
Sprr2A: Small proline-rich protein 2A
TARP: Tissue Array Research Program
TMA: Tissue microarray
V2-R: Arginine vasopressin receptor 2

## Footnotes

1 The Nobel Prize in Chemistry 2003 was awarded “for discoveries concerning channels in cell membranes” jointly with one half to Peter Agre “for the discovery of water channels” and with one half to Roderick MacKinnon “for structural and mechanistic studies of ion channels”.

2
http://www.nobelprize.org/nobel_prizes/chemistry/laureates/2003/
